# Non-Immunologic Actions of Calcineurin Inhibitors in Proteinuric Kidney Diseases

**DOI:** 10.3389/fendo.2014.00181

**Published:** 2014-11-12

**Authors:** Robert Frank Spurney

**Affiliations:** ^1^Division of Nephrology, Department of Medicine, Duke University and Durham VA Medical Centers, Durham, NC, USA

**Keywords:** glomerular podocyte, cell signaling pathways, calcineurin, calcineurin inhibitors, synaptopodin, Rho GTPases

Diseases affecting the glomerulus are the most common cause of end-stage kidney disease in developed countries (1). These disorders are characterized by significant proteinuria, and the level of proteinuria is an independent risk factor for disease progression (2). Podocytes are thought to play a key role in the pathogenesis of glomerular diseases (3, 4). The importance of podocytes in glomerular diseases is highlighted by genetic studies, which have identified mutant podocyte proteins that cause familial forms of nephrosis (5). Because podocytes are terminally differentiated cells with little capacity for replication, their ability to compensate for podocyte loss is limited (3). A component of current therapy is, therefore, focused on reducing podocyte injury by decreasing systemic blood pressure (BP) and inhibition of the renin–angiotensin system (2, 6).

Historically, the immune system was thought to play a significant role in non-genetic forms of nephrosis including acquired diseases such as minimal-change disease (MCD) and focal segmental glomerulosclerosis (FSGS) (7). As a result, corticosteroids and CNIs are often used to treat these disorders (6). Indeed, the response to steroid therapy is an important prognostic indicator for both MCD and FSGS (8, 9). Recent studies, however, suggest that these agents may have actions that are independent of their immunosuppressive properties. For example, while not a universal finding (10, 11), steroids and/or CNIs are reported to induce partial or complete remissions of proteinuria in a subset of patients with genetic forms of nephrosis (7, 11, 12). Although we acknowledge that these reports have significant limitations, the data support the concept that that steroids and/or CNIs may have beneficial effects unrelated to their immunosuppressive actions. Similarly, CNIs inhibit death of cultured podocytes after apoptotic stimuli despite the absence of immune effector mechanisms in the tissue culture model (13, 14). Moreover, genetic activation of the CN effector NFAT (nuclear factor of activated T cells) in podocytes promotes proteinuria, glomerulosclerosis, and a decrease in podocyte numbers in mice despite restricting the experimental manipulation to glomerular podocytes (15).

As shown in Figure 1, non-immunological actions of CNIs can be broadly divided into effects on the podocyte cytoskeleton and effects on podocyte survival. A seminal observation was that the actin-associated protein synaptopodin (SYN) was phosphorylated by either protein kinase A (PKA) or calcium/calmodulin-dependent protein kinase II (CaMKII). Phosphorylation of SYN provided a docking site for 14–3–3 proteins and prevented degradation of SYN by the cysteine proteinase cathepsin L (16). Dephosphorylation of the 14–3–3 docking site by calcium sensitive phosphatase CN promoted SYN degradation. This group further demonstrated that SYN competitively antagonized ubiquitination of Rho A by Smurf1 (SMAD specific E3 ubiquitin protein ligase 1), and promoted Rho A activation and stress fiber formation (17). While Rho A activity is also stimulated by calcium-dependent mechanisms (18, 19), the SYN dependence of these effects appeared relevant to glomerular diseases because expression of a degradation resistant SYN in podocytes protected mice from proteinuric stimuli (16). In this scenario, CNIs promote a podocyte phenotype that is resistant to the development of proteinuria by stabilizing the actin cytoskeleton.

**Figure 1:**
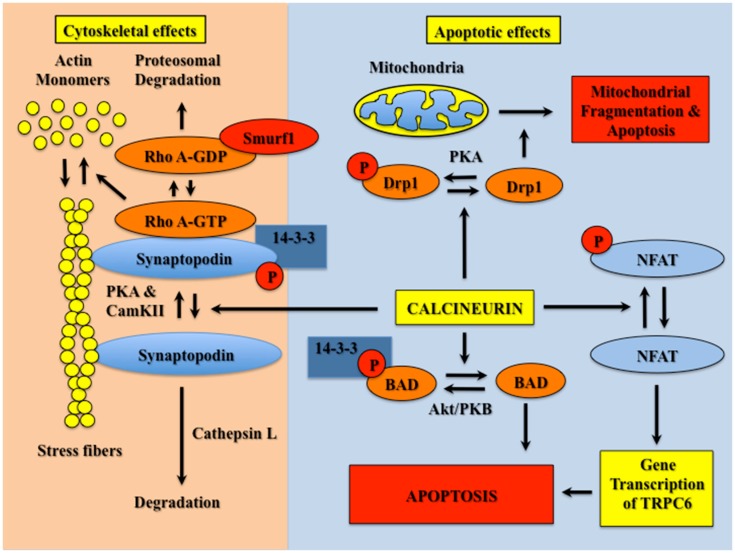
**CN activation destabilizes the actin cytoskeleton and causes podocyte apoptosis**. Phosphorylation of SYN is mediated by PKA and CamKII. Phosphorylated SYN promotes 14–3–3 binding, which protects SYN from degradation by cathepsin L. SYN also binds Rho A, and competitively inhibits binding of Rho A to the ubiquitin ligase Smurf1, which prevents targeting of Rho A for proteasomal degradation. Binding of Rho A to SYN activates Rho A (GTP bound Rho A) and, in turn, induces stress fiber formation and stabilizes the podocyte cytoskeleton. CN dephosphorylates the 14–3–3 docking site in SYN and promotes SYN degradation by cathepsin L. In the absence of SYN, Rho A is targeted for proteosomal degradation, which reduces stress fiber formation and destabilizes the actin cytoskeleton. CN also promotes podocyte apoptosis by dephosphorylation of either NFAT isoforms, Drp1 or BAD. This apoptotic effect is mediated both directly by Drp1- or BAD-dependent activation of mitochondrial apoptotic pathways, as well as indirectly by stimulation of NFAT-dependent gene transcription.

CNIs also protect podocytes from apoptotic stimuli (13, 14). At least one mechanism is dependent on gene transcription induced by NFAT (13, 14). NFAT transcription factors were originally discovered in cells of the lymphoid lineage, but abundant evidence indicates that NFAT isoforms are expressed in non-immune cells with some family members expressed ubiquitously (20). In quiescent cells, NFAT isoforms are phosphorylated and located in the cytoplasm (20). CN dephosphorylates NFAT, which permits translocation to the nucleus and stimulation of gene transcription. In cultured podocytes, expression of a constitutively active CN construct causes apoptosis, and this apoptotic effect is blocked by the pharmacologic CNI FK506 as well as by a peptide inhibitor of CN termed VIVIT (13). Similarly, hyperglycemia induces nuclear localization of NFAT isoforms as well as promotes apoptosis of cultured podocytes, and this apoptotic effect is also attenuated by VIVIT (14). Moreover, CN activity is enhanced in kidneys of diabetic rodents (13, 21), and treatment with FK506 attenuates hyperglycemia-induced podocyte apoptosis in diabetic mice (13). Because VIVIT specifically inhibits CN-dependent NFAT activation (22), these data suggest that CN causes podocyte apoptosis by mechanisms that require NFAT mediated gene transcription. In this regard, TRPC6 (transient receptor potential channel C6) is an important gene target of NFAT transcription factors (23). Indeed, gain-of-function mutations in TRPC6 cause FSGS (24, 25). TRPC6 is also up-regulated in primary glomerular diseases (26) and over-expression of TRPC6 in podocytes causes proteinuric kidney disease (27). Thus, TRPC6 may be an important downstream gene target of CN signaling in glomerular disorders. In contrast, one study reported that CNIs induced podocyte apoptosis (28). This report, however, is controversial, and we and others (7) have not been able to reproduce this observation.

As shown in Figure 1, other mechanisms of CN-mediated podocyte apoptosis include induction of mitochondrial fragmentation by Drp1 (dynamin related protein 1) as well as activation of the apoptosis inducing Bcl-2 family member BAD (Bcl-2 associated death promoter). Drp1 is phosphorylated and inhibited by PKA (29); BAD is phosphorylated by Akt, which causes sequestration of BAD by 14–3–3 proteins and inhibits apoptosis (30). CN dephosphorylates both proteins and induces apoptotic cell death through the mitochondrial pathway (29, 30). Both Drp1 and BAD have been implicated in the pathogenesis of glomerular diseases by promoting podocyte apoptosis (31–33), with the extent of apoptosis presumably dependent on the relative activities of CN and the relevant kinases. Based on these observations as well as the NFAT-dependent apoptotic effects described above, we speculate that CNIs might be useful therapies for attenuating podocyte apoptosis in diseases with either enhanced CN activity or in diseases associated with reduced activity of the relevant kinase.

In summary, CNIs may have important beneficial effects for both the podocyte cytoskeleton and podocyte viability. These agents attenuate podocyte apoptosis as well as promote a podocyte phenotype that is resistant to the development of proteinuria. The beneficial effects of CNIs may be mediated by mechanisms that are independent of the immune system. Given the potential role of CN in diverse glomerular diseases, the use of CNIs might be useful for a broader range of kidney disorders. We acknowledge that CNI nephrotoxicity is a concern (34), but the development of more specific agents with fewer off-target effects (35, 36) may be an effective strategy for expanding the use of CN inhibition to a broader range of glomerular disease processes.

## Conflict of Interest Statement

The author declares that the research was conducted in the absence of any commercial or financial relationships that could be construed as a potential conflict of interest.
